# Rural and urban differences in patient experience in China: a coarsened exact matching study from the perspective of residents

**DOI:** 10.1186/s12913-021-06328-0

**Published:** 2021-04-13

**Authors:** Dantong Zhao, Zhongliang Zhou, Chi Shen, Rashed Nawaz, Dan Li, Yangling Ren, Yaxin Zhao, Dan Cao, Xiaohui Zhai

**Affiliations:** 1 School of Public Policy and Administration, Xi’an Jiaotong University, No. 28 Xianning West Road, Xi’an, 710049 Shaanxi China; 2 Department of Health Policy and Management, Yale University, New Haven, CT 06520 USA; 3 School of Public Health, Health Science Center, Xi’an Jiaotong University, Xi’an, 710061 China

**Keywords:** Patient experience, Rural and urban differences, China, Coarsened exact matching

## Abstract

**Background:**

Patient experience is a key measure widely used to evaluate quality of healthcare, yet there is little discussion about it in China using national survey data. This study aimed to explore rural and urban differences in patient experience in China.

**Methods:**

Data regarding this study were drawn from Chinese General Social Survey (CGSS) 2015, with a sample size of 9604. Patient experience was measured by the evaluation on healthcare services. Coarsened exact matching (CEM) method was used to balance covariates between the rural and urban respondents. Three thousand three hundred seventy-two participants finally comprised the matched cohort, including 1592 rural residents and 1780 urban residents. Rural and urban differences in patient experience were tested by ordinary least-squares regression and ordered logistic regression.

**Results:**

The mean (SD) score of patient experience for rural and urban residents was 72.35(17.32) and 69.45(17.00), respectively. Urban residents reported worse patient experience than rural counterparts (Crude analysis: Coef. = − 2.897, 95%CI: − 4.434, − 1.361; OR = 0.706, 95%CI: 0.595, 0.838; Multivariate analysis: Coef. = − 3.040, 95%CI: − 4.473, − 1.607; OR = 0.675, 95%CI: 0.569, 0.801). Older (Coef. = 2.029, 95%CI: 0.338, 3.719) and healthier (Coef. = 2.287, 95%CI: 0.729, 3.845; OR = 1.217, 95%CI: 1.008, 1.469) rural residents living in western area (Coef. = 2.098, 95%CI: 0.464, 3.732; OR = 1.276, 95%CI: 1.044, 1.560) with higher social status (Coef. = 1.158, 95%CI: 0.756, 1.561; OR = 1.145, 95%CI: 1.090, 1.204), evaluation on adequacy (Coef. = 7.018, 95%CI: 5.045, 8.992; OR = 2.163, 95%CI: 1.719, 2.721), distribution (Coef. = 4.464, 95%CI: 2.471, 6.456; OR = 1.658, 95%CI: 1.312, 2.096) and accessibility (Coef. = 2.995, 95%CI: 0.963, 5.026; OR = 1.525, 95%CI: 1.217, 1.911) of healthcare resources had better patient experience. In addition, urban peers with lower education (OR = 0.763, 95%CI: 0.625, 0.931) and higher family economic status (Coef. = 2.990, 95%CI: 0.959, 5.021; OR = 1.371, 95%CI: 1.090,1.723) reported better patient experience.

**Conclusions:**

Differences in patient experience for rural and urban residents were observed in this study. It is necessary to not only encourage residents to form a habit of seeking healthcare services in local primary healthcare institutions first and then go to large hospitals in urban areas when necessary, but also endeavor to reduce the disparity of healthcare resources between rural and urban areas by improving quality and capacity of rural healthcare institutions and primary healthcare system of China.

**Supplementary Information:**

The online version contains supplementary material available at 10.1186/s12913-021-06328-0.

## Background

Feedback of equipment, environment and services from healthcare users, is of vital importance to quality assessment and healthcare services improvement [1]. As a crucial measure widely utilized to evaluate quality of healthcare [2, 3], patient experience has been increasingly adopted by researchers, regulators, and policy makers [4–9]. Previous researches demonstrated that better patient experience was positively associated with better health outcomes and higher levels of adherence to prevention, medication and treatment processes recommended by physicians [10], as well as less adverse events and healthcare utilization [11].

Globally there is an increasing trend towards research on patient experience. The growing focus on patient experience reflects the fact of broader emphases and actions on patient-centered healthcare delivery [12]. The measure of patient experience is applied to public reports and pay-for-performance programs in healthcare systems [13]. As early as 2001, The National Health Service (NHS) in the UK conducted a pioneering national patient survey [14], followed by the USA with its The Hospital Consumer Assessment of Healthcare Providers and Systems (HCAHPS) survey in 2006 [15]. By comparison, China should make efforts to conduct representative and national patient experience survey, and then release the authoritative reports on the patient experience survey results.

Patient experience is a multi-dimensional construct encompassing numerous elements of healthcare. It can be measured by either patient report (what happened to patient) or patient evaluation (how patients rate) items, including the process of an appointment, waiting time, cleanliness of environment, efficiency of apparatus, communication with doctors and nurses, responsiveness and interactions with medical staffs and receptionists, and so on [4]. Albeit several studies measured patient experience and some regional surveys were conducted in China [13, 16–18], large-scale and national collecting data on patient experience was still scarce [19], neither rural-urban status quo nor rural-urban differences in patient experience were mentioned among Chinese population.

Rural and urban disparities were embodied in various aspects. Compared with their urban counterparts, rural residents are more likely to be unemployed with lower level of education and income [20, 21], and China is no exception [22]. Furthermore, lower utilization of healthcare services is not uncommon in rural areas of many countries [23–26]. Nowadays, in spite of stable economic growth and improved health status of residents in both rural and urban areas in China, there is still noticeable urban-rural inequality in health-related issues, such as healthcare utilization [27, 28], healthcare resources [29], access to healthcare services [30, 31] and prevalence of certain diseases [32–34]. Urban residents are likely to have more healthcare utilization, more healthcare resources (including institutions, beds and health professionals), more access to government sponsored public programs/ healthcare services and less risk to suffer from disease, such as breast cancer, depression and chronic diseases (i.e., hypertension, chronic ischemic heart disease, cerebrovascular disease and arthritis). Based on differences in rural and urban residents’ socioeconomic and health-related issues, it may result in their different patient experiences during healthcare services utilization and which may also be explained by different factors [19]. Thus, it is very essential to explore whether there are discrepant patient experiences between these two groups or not, and further examine the potential influencing factors of their patient experiences, respectively.

**Fig. 1:**
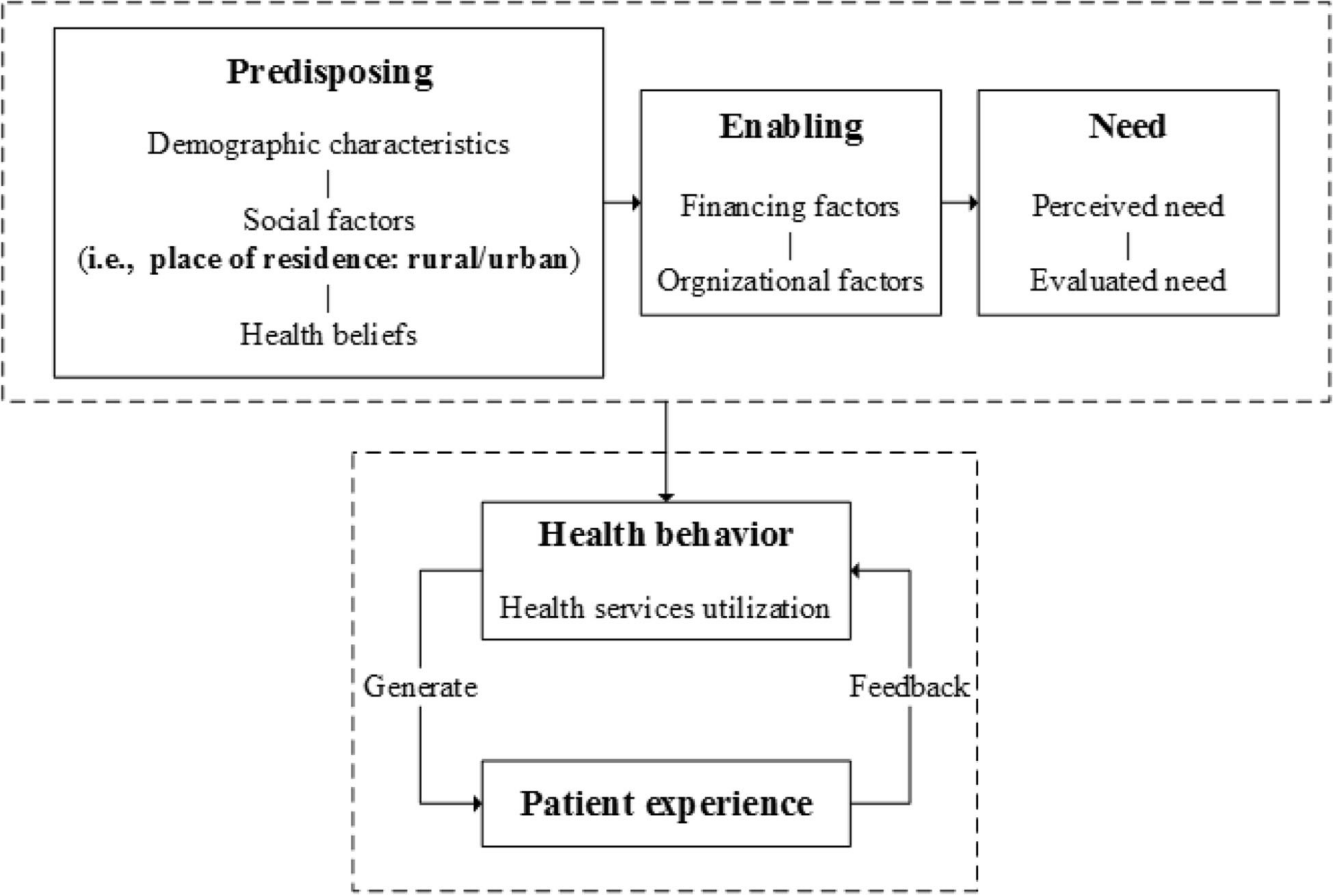
Conceptual framework for patient experience based on Andersen’s Behavioral Model of Health Services Use

On the concept of theory of Andersen’s Behavioral Model of Health Services Use [35–37], patient experience is regarded as feedback of healthcare utilization behavior, and it can be explained by predisposing, enabling and need factors. As an indicator of predisposing factors, place of residence distinguishes the rural and the urban. Figure 1 showed the conceptual framework for patient experience based on Andersen’s Behavioral Model of Health Services Use. Coarsened exact matching (CEM) method, firstly put forward by Iacus, et al. [38, 39], aims to better balance the multidimensional distribution of covariates between the two comparison groups, and thereby reduces the degree of dependence on estimation model of the dependent variable and further decreases the biases. Previous studies demonstrated that both matched sampling and regression adjustment could be expected to better reduce biases [40], and matching method application was more robust than regression analysis alone [41]. Considering only regression model was used to control the confounders in previous studies on patient experience [5, 6], the combination of the CEM method and the regression model were applied in this study.

A key issue arises from the described background: there is little discussion about patient experience differences between comparable rural and urban residents in China by using a national survey data under the theory of Andersen’s Behavioral Model of Health Services Use. The focus of current study was rural and urban differences in patient experience in China, and the objectives were: 1) to explore the association between place of residence and patient experience; 2) to examine the factors affecting patient experience for rural and urban residents, respectively.

## Methods

### Theory of Andersen’s behavioral model of health services use

Based on the theory of Andersen’s Behavioral Model of Health Services Use, an individual’s utilization of healthcare service is a behavior of his/her need for such healthcare service, a predisposition to use them and factors that enable them to be utilized [42]. Generally speaking, predisposing factors include the demographic characteristics of age and gender, social factors, such as ethnicity/nativity, education, marital status, social status, the place of residence and health beliefs (i.e., knowledge, attitudes, value elated to health and health services); enabling factors refers to income/financial situation, medical insurance, usual source of care, availability and accessibility of healthcare services; need factors are evaluated health status, perceived need and self-rated health [43, 44].

### Data source

Data of this study were drawn from Chinese General Social Survey (CGSS) 2015, a national, representative and all-around survey first conducted in 2003 by Renmin University of China (RUC) and Hong Kong University of Science and Technology. CGSS can be considered as the Chinese counterpart of the General Social Survey (GSS) in the US [45]. A multi-stage stratified random sampling method was employed and populations in both rural and urban China were surveyed. CGSS 2015 covered 478 villages from 28 provinces/municipalities and contained 10,968 valid samples. The questionnaire consisted of 6 parts (Part A–F) corresponding to different contents, where Part A and B investigated all samples, Part C and D investigated 1/6 of all samples and Part E and F only covered 1/3. The data exploited in this study were Part A and B, which included most variables related to social life and health services. Nine thousand six hundred four participants were finally included after the missing values were withdrawn.

### Outcome and covariate variables

As a primary outcome variable, patient experience, regarded as feedback of healthcare utilization, was measured by healthcare services evaluation with following question: “What score will you give to healthcare services (0–100)?” In addition, during the analysis, the 0–100 values were further divided into five ordered levels: very bad (value = 0), bad (value< 60), fair (value ranges from 60 to 79), good (value ranges from 80 to 99), very good (value = 100), coding from 1 to 5. Ordered classification was used to better depict participants’ subjective perception, since Chinese are accustomed to assessing the quality by dividing centesimal system into such levels.

Based on the concept of Andersen’s Behavioral Model of Health Services Use, the evaluation on healthcare services could be explained by independent variables as follows: 1) predisposing factors: age, gender, education, marital status, social status, place of residence and region; 2) enabling factors: personal income, family economic status, medical insurance, the evaluation on adequacy, distribution and accessibility of healthcare resources; 3) need factors: self-rated health status. Social status was measured by the following question: “What level/layer do you think you are in society?”, with an answer from value 1 to 10 and higher value indicating higher social class. Thus, the key independent variable was place of residence classified by sample type (rural/urban; a dichotomous variable). Control variables included age (below 45, 46–59 and above 60 years old; a categorical variable), gender (male/female; a dichotomous variable), education (primary school and below, junior and senior school, college degree and above; a categorical variable), marital status (married/others [including those who were unmarried, divorced, widowed and cohabiting]; a dichotomous variable), social status (1–10; a continuous variable), and region (Eastern, Middle and Western; a categorical variable), personal income (in quintiles: poorest, 2nd, middle, 4st and richest; a categorical variable), family economic status (lower than average, average and higher than average; a categorical variable), medical insurance (no/yes; a dichotomous variable), the evaluation on adequacy (inadequate, fair and adequate; a categorical variable), distribution (unbalanced, fair and balanced; a categorical variable) and accessibility of healthcare resources (inconvenient, fair and convenient; a categorical variable), self-rated health status (unhealthy, fair and healthy; a categorical variable). For further information about the definitions/codes of variables, please check the supplementary files (Table S1).

### Coarsened exact matching method

CEM is a matching method of the class Monotonic Imbalance Bounding (MIB), which shows the basic advantage over other matching methods that reducing imbalance in the empirical distribution in one covariate won’t affect any other covariates chosen for balancing [41]. Furthermore, it is preferable to other matching procedures (i.e., propensity score matching) in terms of processing more efficiently and reducing model dependence, variance and bias applied in contemporary health and epidemiological research [46, 47]. In general, CEM algorithm has three procedures. First of all, each variable is coarsened by recoding, and thereby indistinguishable values are grouped and appointed the same numerical value. Secondly, the coarsened data are matched by the algorithm of exact matching, and then unmatched units are pruned. Thirdly, the coarsened data are removed and the uncoarsened values of the matched data are retained. Additionally, a weighting variable is generated by CEM method to equalize the number of observations within comparison groups, ranging from 0 to 1 [39]. For balance checking of two comparison groups, the multivariate imbalance measure L_1_ is employed, of which size depends on the data set and the selected covariates. L_1_ ranges from 0 to 1, where 0 and 1 means perfect global balance and maximal imbalance respectively, and larger value represents larger imbalance between two groups. A good matching usually brings a substantial reduction in L_1_ [48]. In our study, CEM was used to make the two comparison groups of rural and urban residents statistically equivalent during examination of the relationship between place of residence and patient experience, based on age, gender, education status, marital status, social status, region, personal income, family economic status, medical insurance and self-rated health status.

### Statistical analysis

Chi-square test for categorical variables and one-way ANOVA for continuous variables were used to examine whether the rural-urban difference was statistically significant or not. Ordered logistic regression for ordinal categorical outcome and ordinary least-squares regression for numerical outcome were applied to examine the association between place of residence and patient experience in the matched cohort, and to explore factors affecting the patient experience for rural and urban residents in the unmatched cohort with sample weights. In matched data, all analyses were conducted incorporating matched weights. Potential confounders were controlled in multivariate analysis. All analyses were performed by using Stata software (version 15.0; StataCorp). All statistical tests were two sided with a significance threshold of 0.05.

## Results

**Fig. 2:**
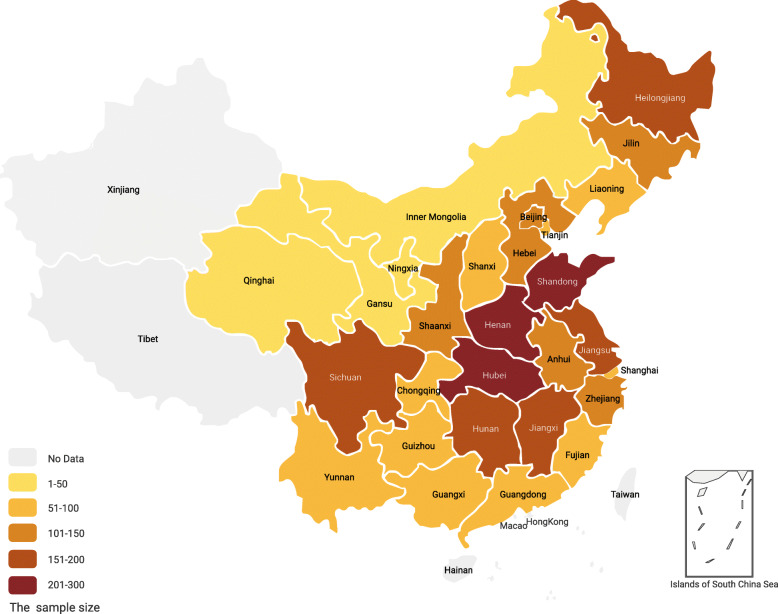
The sample distribution. Note: The map was generated by using Figma Desktop APP version 95.8

Figure 2 presented sample distribution. The multivariate imbalance measure L_1_ statistic before and after CEM was reported in Table 1. After matching, the L_1_ was reduced from 0.771 to 6.504e-16 and all matched variables after CEM were also close to zero. The results indicated that the rural and the urban became more comparable and balanced after matching process.

**Table 1:** The L_1_ statistic before and after coarsened exact matching method (CEM)

Variables	Before Matching (*N* = 9604)	After Matching (*N* = 3372)
	L_1_(mean)	L_1_(mean)
Age	0.110(− 0.152)	1.7e-15 (3.3e-15)
Gender	0.022(0.022)	3.4e-15 (2.4e-15)
Education status	0.334(0.557)	2.4e-15 (− 6.7e-15)
Marital status	0.059(−0.059)	4.4e-16 (2.2e-16)
Social status	0.098(0.357)	1.2e-15(2.0e-14)
Region	0.325(−0.497)	9.2e-16(1.4e-14)
Personal income	0.070(0.013)	4.2e-16(4.4e-15)
Family economic status	0.059(0.106)	1.8e-15(−2.9e-15)
Medical insurance	0.023(−0.023)	9.5e-18(0)
Self-rated health status	0.107(0.192)	6.7e-16(8.0e-15)
Multivariate L_1_	0.771	6.504e-16

Note: The mean is labeled in parentheses and reports the difference in means

**Fig. 3:**
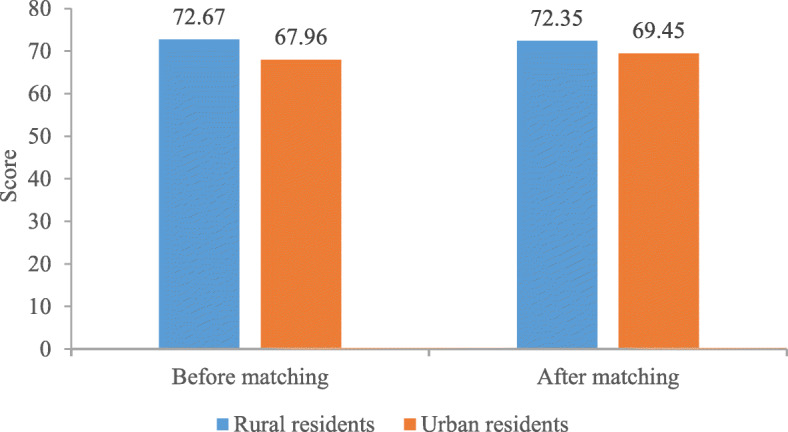
Comparison of patient experience for urban and rural residents by using numerical outcome variable

**Fig. 4:**
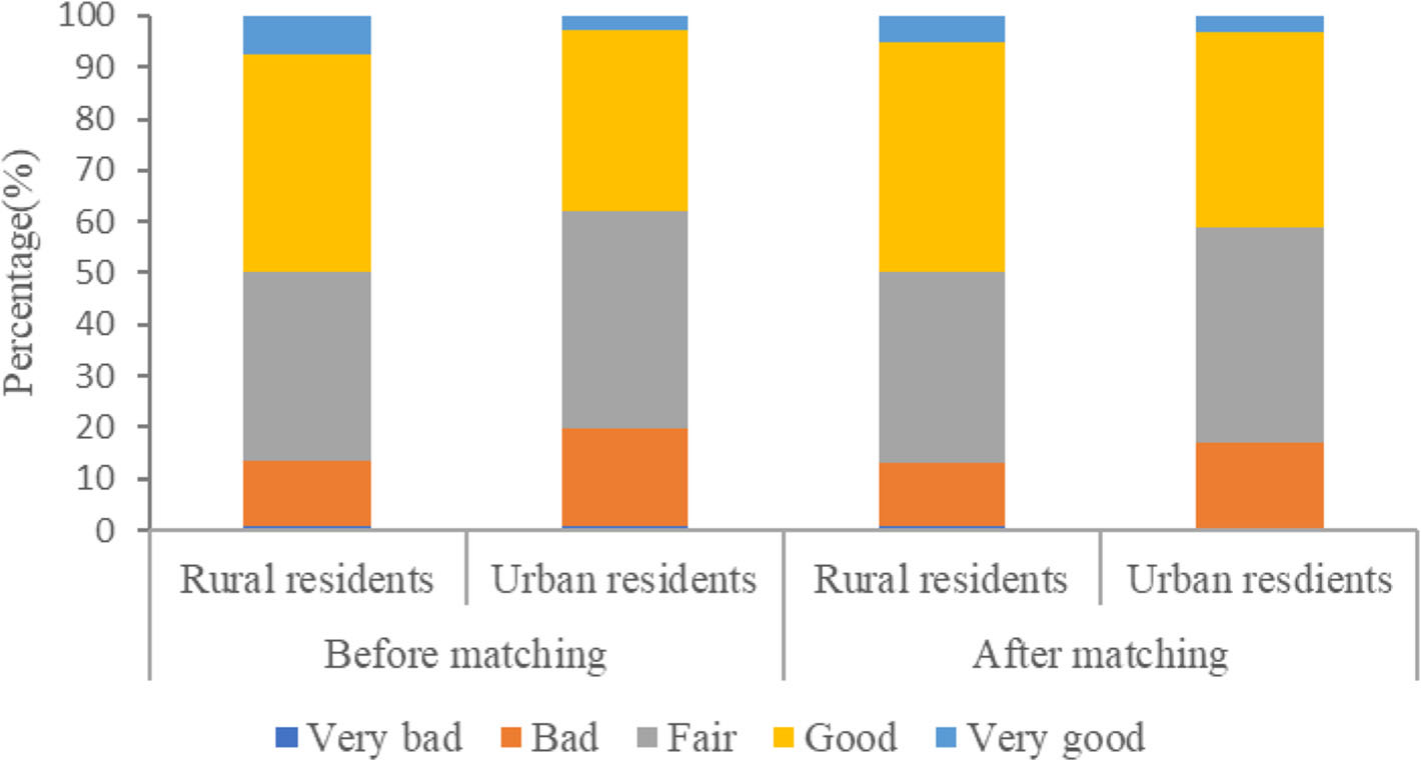
Comparison of patient experience for urban and rural residents by using ordinal categorical outcome variable

Patient experience and basic characteristics of rural and urban residents before and after matching were presented in Table 2. The unmatched cohort consisted of 3914 rural residents and 5690 urban residents, and most characteristics of rural and urban residents were significantly different. After improving balance between two comparison groups, 1592 rural residents and 1780 urban residents comprised the matched cohort. No statistical difference was found in any characteristic of rural and urban residents, except for personal income. Of the 3372 matched rural and urban residents, half of them were females (54.04%), married (87.47%), aged 60 years and below (75.78%), and had an education of junior and senior school (58.03%). Minority of them lived in western (19.55%) with average family economic (69.27%) and medical insurance (98.60%). In terms of adequacy, distribution and accessibility of healthcare resources, nearly half of respondents thought they were adequate (44.82% vs. 44.83%), balanced (34.01% vs. 36.46%) and convenient (44.80% vs. 45.39%). Majority of respondents were healthy (76.69%). As presented in Table 2, Fig. 3 and Fig. 4, rural residents always had better patient experience than their urban counterparts before and after matching.

**Table 2:** Basic characteristics of rural and urban residents in unmatched and matched cohort

Variables	Unmatched			Matched		
	Rural	Urban	*p*-value	Rural	Urban	*p*-value^3^
N	3914	5690		1592	1780	
Patient experience^a,1^			< 0.001			< 0.001
Very bad	32(0.82)	35(0.62)		14(0.86)	8(0.45)	
Bad	501(12.80)	1098(19.30)		197(12.35)	294(16.52)	
Fair	1430(36.54)	2388(41.97)		589(36.98)	746(41.91)	
Good	1652(42.21)	2001(35.17)		708(44.50)	677(38.03)	
Very good	299(7.64)	168(2.95)		84(5.31)	55(3.09)	
Patient experience^b,2^	72.67(17.55)	67.96(17.82)	< 0.001	72.35(17.32)	69.45(17.00)	< 0.001
Age^1^ (years)			< 0.001			1.000
≤ 45	1298(33.16)	2514(44.18)		723(45.39)	808(45.39)	
46–60	1361(34.77)	1589(27.93)		484(30.39)	541(30.39)	
> 60	1255(32.06)	1587(27.89)		385(24.21)	431(24.21)	
Gender^1^			0.038			0.988
Male	1923(49.13)	2673(46.98)		732(45.96)	818(45.96)	
Female	1991(50.87)	3017(53.02)		860(54.04)	962(54.04)	
Education status^1^			< 0.001			1.000
Primary school and below	2195(56.08)	1290(22.67)		491(30.84)	549(30.84)	
Junior and senior school	1580(40.37)	2931(51.51)		924(58.03)	1033(58.03)	
College degree and above	139(3.55)	1469(25.82)		177(11.12)	198(11.12)	
Marital status^1^			< 0.001			0.980
Unmarried/ Divorced/ Widowed/ Cohabiting	725(18.52)	1392(24.46)		199(12.53)	223(12.53)	
Married	3189(81.48)	4298(75.54)		1393(87.47)	1557(87.47)	
Social status^2^	4.12(1.66)	4.48(1.59)	< 0.001	4.54(1.20)	4.54(1.20)	1.000
Region^1^			< 0.001			1.000
Eastern	797(20.36)	3007(52.85)		638(40.06)	713(40.06)	
Middle	1765(45.09)	1696(29.81)		643(40.39)	719(40.39)	
Western	1352(34.54)	987(17.35)		311(19.55)	348(19.55)	
Personal income^1^			< 0.001			< 0.001
Poorest	787(20.11)	1138(20.00)		361(22.69)	364(20.45)	
2nd	805(20.57)	1216(21.37)		292(18.37)	433(24.33)	
Middle	920(23.513)	1067(18.75)		343(21.53)	317(17.81)	
4st	653(16.68)	1304(22.92)		322(20.22)	360(20.22)	
Richest	749(19.14)	965(16.96)		274(17.19)	306(17.19)	
Family economic status^1^			< 0.001			0.999
Lower than average	1592(40.67)	1976(34.73)		418(26.29)	468(26.29)	
Average	2090(53.40)	3109(54.64)		1103(69.27)	1233(69.27)	
Higher than average	232(5.93)	605(10.63)		71(4.44)	79(4.44)	
Medical insurance^1^			< 0.001			0.955
No	244(6.23)	486(8.54)		22(1.40)	25(1.40)	
Yes	3670(93.77)	5204(91.46)		1570(98.60)	1755(98.60)	
Evaluation on adequacy of healthcare resources^1^			0.892			0.908
Inadequate	1118(28.56)	1606(28.22)		435(27.32)	476(26.74)	
Fair	1074(27.44)	1584(27.84)		444(27.86)	506(28.43)	
Adequate	1722(44.00)	2500(43.94)		713(44.82)	798(44.83)	
Evaluation on distribution of healthcare resources^1^			0.028			0.606
Unbalanced	1338(34.18)	2095(36.82)		552(34.68)	609(34.21)	
Fair	1164(29.74)	1643(28.88)		498(31.31)	522(29.33)	
Balanced	1412(36.08)	1952(34.31)		542(34.01)	619(36.46)	
Evaluation on accessibility of healthcare resources^1^			0.948			0.878
Inconvenient	1153(29.46)	1669(29.33)		442(27.29)	498(27.98)	
Fair	1033(26.39)	1490(26.19)		436(27.41)	474(26.63)	
Convenient	1728(44.15)	2531(44.48)		713(44.80)	808(45.39)	
Self-rated health status ^1^			< 0.001			0.999
Unhealthy	930(23.76)	742(13.04)		165(10.34)	184(10.34)	
Fair	791(20.21)	1280(22.50)		207(12.98)	231(12.98)	
Healthy	2193(56.03)	3668(64.46)		1220(76.69)	1365(76.69)	

Note: N (%) or Mean (SD) are reported

^a^ Measured by numerical variable

^b^ Measured by ordinal categorical variable

^1^ Chi-square test

^2^ One-way ANOVA

^3^ Considering match weights

Table 3 illustrated the association between place of residence and patient experience in the matched cohort with CEM weights by using ordinary least-squares regression and ordered logistic regression, respectively. Both crude and multivariate analysis showed urban residents reported worse patient experience than rural counterparts (Crude: Coef. = − 2.897, 95%CI: − 4.434, − 1.361; OR = 0.706, 95%CI: 0.595, 0.838; Multivariate regression: Coef. = − 3.040, 95%CI: − 4.473, − 1.607; OR = 0.675, 95%CI: 0.569, 0.801). In order to validate the robustness of results, we employed the regression using the unmatched sample with sample weights (Table S2). We found that urban residents still had lower patient experience compared to rural residents.

**Table 3:** The association between place of residence and patient experience in the matched cohort with CEM weights

Variables	Crude analysis		Multivariate analysis	
	Coef.^a^	OR^b^	Coef.^c^	OR^d^
	95%CI	95%CI	95%CI	95%CI
Place of residence (Ref: Rural)				
Urban	−2.897^***^	0.706^***^	−3.040^***^	0.675^***^
	(−4.434 - -1.361)	(0.595–0.838)	(−4.473 - -1.607)	(0.569–0.801)
Control variables	No	No	Yes	Yes

Note: Coef. means Coefficient. OR means Odds Ratio. 95%CI means 95% Confidence Interval

^*^ *p* < 0.05, ^**^ *p* < 0.01, ^***^ *p* < 0.001

^a^ Crude ordinary least-squares regression

^b^ Crude ordered logistic regression

^c^ Ordinary least-squares regression within the control of age, gender, education, marital status, social status, region, personal income, family economic status, medical insurance, the evaluation on adequacy, distribution and accessibility of healthcare resources, and self-rated health status

^d^ Ordered logistic regression within the control of age, gender, education, marital status, social status, region, personal income, family economic status, medical insurance, the evaluation on adequacy, distribution and accessibility of healthcare resources, and self-rated health status

Table 4 displayed the factors affecting patient experience for rural and urban residents in the unmatched cohort with sample weights. Older (Coef. = 2.029, 95%CI: 0.338, 3.719), female (Coef. = 1.292, 95%CI: 0.064, 2.519) and healthier (Coef. = 2.287, 95%CI: 0.729, 3.845; OR = 1.217, 95%CI: 1.008, 1.469) rural residents living in western area (Coef. = 2.098, 95%CI: 0.464, 3.732; OR = 1.276, 95%CI: 1.044, 1.560) with higher social status (Coef. = 1.158, 95%CI: 0.756, 1.561; OR = 1.145, 95%CI: 1.090, 1.204), evaluation on adequacy (Coef. = 7.018, 95%CI: 5.045, 8.992; OR = 2.163, 95%CI: 1.719, 2.721), distribution (Coef. = 4.464, 95%CI: 2.471, 6.456; OR = 1.658, 95%CI: 1.312, 2.096) and accessibility (Coef. = 2.995, 95%CI: 0.963, 5.026; OR = 1.525, 95%CI: 1.217, 1.911) of healthcare resources had better patient experience. By comparison, in addition to the same association between age, health status, social status, living region, evaluation on adequacy, distribution and accessibility of healthcare resources and patient experience as rural residents, urban peers with lower education (OR = 0.763, 95%CI: 0.625, 0.931) and higher family economic status (Coef. = 2.990, 95%CI: 0.959, 5.021; OR = 1.371, 95%CI: 1.090,1.723) had better patient experience.

**Table 4:** Factors affecting patient experience for rural and urban residents in the unmatched cohort

Variables	Rural		Urban	
	Coef.^a^	OR^b^	Coef.^a^	OR^b^
	95%CI	95%CI	95%CI	95%CI
Age, years (Ref: ≤45)				
46–60	1.487	1.192	1.231	1.115
	(− 0.054–3.028)	(0.998–1.424)	(− 0.085–2.547)	(0.965–1.288)
> 60	2.029^*^	1.119	2.314^**^	1.229^**^
	(0.338–3.719)	(0.915–1.369)	(0.919–3.710)	(1.052–1.437)
Gender (Ref: Male)				
Female	1.292^*^	1.130	0.257	1.040
	(0.064–2.519)	(0.975–1.310)	(− 0.770–1.283)	(0.927–1.167)
Education status (Ref: Primary school and below)				
Junior and senior school	− 0.331	0.894	− 1.099	0.841^*^
	(−1.694–1.033)	(0.760–1.053)	(− 2.530–0.332)	(0.715–0.989)
College degree and above	−0.428	0.918	−1.355	0.763^**^
	(−3.318–2.463)	(0.652–1.292)	(−3.140–0.430)	(0.625–0.931)
Marital status (Ref: Unmarried/Divorced/Widowed/Cohabiting)				
Married	−0.484	1.003	−0.664	0.938
	(−1.982–1.014)	(0.838–1.200)	(−1.850–0.523)	(0.821–1.072)
Social status	1.158^***^	1.145^***^	0.955^***^	1.101^***^
	(0.756–1.561)	(1.090–1.204)	(0.573–1.337)	(1.056–1.148)
Region (Ref: Eastern)				
Middle	0.0198	1.103	2.847^***^	1.481^***^
	(−1.565–1.605)	(0.914–1.330)	(1.620–4.075)	(1.291–1.698)
Western	2.098^*^	1.276^*^	3.947^***^	1.570^***^
	(0.464–3.732)	(1.044–1.560)	(2.559–5.335)	(1.331–1.852)
Personal income (Ref: Poorest)				
2nd	1.748	1.168	− 0.466	0.956
	(−0.045–3.541)	(0.942–1.449)	(− 2.089–1.156)	(0.794–1.151)
Middle	0.350	1.039	−1.447	0.795^*^
	(−1.509–2.209)	(0.836–1.291)	(−3.086–0.193)	(0.660–0.957)
4st	0.807	1.056	−0.158	1.016
	(−1.175–2.788)	(0.834–1.338)	(−1.841–1.526)	(0.842–1.225)
Richest	0.202	1.014	−2.129^*^	0.803^*^
	(−1.831–2.235)	(0.797–1.289)	(−3.996 - -0.261)	(0.650–0.992)
Family economic status (Ref: Lower than average)				
Average	0.341	0.974	1.908^**^	1.187^*^
	(−0.962–1.643)	(0.832–1.141)	(0.699–3.116)	(1.039–1.358)
Higher than average	−0.435	0.867	2.990^**^	1.371^**^
	(−2.820–1.950)	(0.644–1.166)	(0.959–5.021)	(1.090–1.723)
Medical insurance (Ref: No)				
Yes	1.892	1.158	0.869	1.107
	(−0.611–4.395)	(0.871–1.539)	(−1.058–2.795)	(0.893–1.373)
Evaluation on adequacy of healthcare resource (Ref: Inadequate)				
Fair	2.963^**^	1.441^***^	3.178^***^	1.497^***^
	(1.062–4.864)	(1.160–1.791)	(1.581–4.775)	(1.260–1.779)
Adequate	7.018^***^	2.163^***^	7.116^***^	2.345^***^
	(5.045–8.992)	(1.719–2.721)	(5.357–8.875)	(1.922–2.861)
Evaluation on distribution of healthcare resource (Ref: Unbalanced)				
Fair	2.410^**^	1.157	1.672^*^	1.133
	(0.619–4.202)	(0.942–1.422)	(0.202–3.142)	(0.964–1.331)
Balanced	4.464^***^	1.658^***^	3.201^***^	1.594^***^
	(2.471–6.456)	(1.312–2.096)	(1.428–4.975)	(1.313–1.935)
Evaluation on accessibility of healthcare resource (Ref: Inconvenient)				
Fair	0.491	1.129	3.497^***^	1.331^***^
	(−1.411–2.392)	(0.921–1.384)	(1.958–5.035)	(1.125–1.573)
Convenient	2.995^**^	1.525^***^	5.783^***^	1.719^***^
	(0.963–5.026)	(1.217–1.911)	(4.188–7.377)	(1.440–2.053)
Self-rated health status (Ref: Unhealthy)				
Fair	2.121^*^	1.231	1.780	1.114
	(0.370–3.873)	(0.998–1.520)	(−0.164–3.725)	(0.902–1.376)
Healthy	2.287^**^	1.217^*^	3.243^***^	1.313^**^
	(0.729–3.845)	(1.008–1.469)	(1.423–5.063)	(1.076–1.601)

Note: Coef. means Coefficient. OR means Odds Ratio. 95% CI means 95% Confidence Interval

Sample weights are considered

^*^ *p* < 0.05, ^**^ *p* < 0.01, ^***^ *p* < 0.001

^a^ Ordinary least-squares regression

^b^ Ordered logistic regression

## Discussion

The present study explored the relationship between place of residence and patient experience by using national survey data, based on which, further examined the factors affecting patient experience for rural and urban residents, respectively. Our findings revealed that: 1) urban residents had worse patient experience than rural residents; 2) Older and healthier residents living in western area with higher social status, evaluation on adequacy, distribution and accessibility of healthcare resources had better patient experience; 3) in addition, urban residents with lower education and higher family economic status had better patient experience.

In line with previous study [1], we found rural residents were more likely to report better experience in healthcare service utilization than urban residents in China. With the public hospital reform as one of core reforming fields, Chinese Health system reform initiated in 2009, aiming to guarantee universal coverage of essential healthcare and provide secure, efficient, convenient and affordable basic healthcare services [49, 50]. Despite all kinds of policies and considerable substantial investments and talent supports to primary healthcare institutions, large secondary and tertiary public hospitals continue to dominate China’s healthcare delivery [51, 52]. Patients in China often choose to visit higher level hospitals directly and bypass primary healthcare facilities, leading to overcrowding at large secondary and tertiary public hospitals but underutilization of primary care [53]. Such behaviors are mostly the results of public’s low trust in the quality of health services delivered by local primary healthcare institutions [54]. It was reported that in 2015, the number of health technicians per thousand persons in urban was 10.21, nearly 2.62 times more than those in rural areas in China, and that the number of hospital beds per thousand persons in urban and rural areas was respectively 8.27 and 3.71 [55]. Based on obvious disparities in healthcare resources between rural and urban areas, it’s comprehensible that rural residents report worse experiences and urban patients report better when they visit their local healthcare institutions, respectively. Whereas, regarding to urban residents’ worse patient experiences found in this study, it’s more likely to consider that it is rural residents’ crowding to large-scale and high level hospitals in urban area that leads to urban residents’ unpleasant experiences, since it’s less likely for urban patients to visit hospitals located in rural area under the background of their better healthcare resources. Thus, over-crowding, long waiting time, and brief encounters with the physicians gradually and naturally generate at such hospitals in urban China [29]. It was evidenced that over-crowding of hospital was associated with poorer patient experience during encounters with physicians [13]. It is of vital importance to not only change the residents’ willingness to large-scale hospital by health education, but also reduce the disparity of healthcare resources between rural and urban areas as much as possible by improving quality and capacity of rural healthcare institutions and primary healthcare system of China, both in infrastructure and in human resource. Only when primary healthcare institutions do act as gatekeeper will the orderly healthcare seeking behavior gradually form.

It was found that older and healthier residents tended to have better patient experience, consistent with other studies [19, 56]. Potential explanations suggested are that these older individuals are more accepting than the young. Expectations could account for the relationship between self-rated health and patient experience. Evidence demonstrates that patients’ illness condition influences their expectations [57]. Patient with better health status may be less anxious and have lower doctor pre-consultation expectations, and thus they are less likely to experience emotional gap. Regarding social status’s positive association with patient experience, it could be explained by patient’s occupation and economy level. The unemployed and farmers are more likely to report a worse experience during the hospital visit in China [19]. Moreover, residents living in western China were more likely to have good experience during the visit in hospital than their eastern counterparts, which may due to inequality in healthcare resources across areas. In 2015, the number of health technicians per thousand persons in eastern and western was 11.0 and 9.4 in urban, and 4.2 and 4.0 in rural [55], which suggested more adequate healthcare resources and higher healthcare quality in eastern area. Based on patients’ preferences to utilize health services with high quality, those who live in western China may trend to visit hospitals located in eastern area for healthcare seeking. Then, over-crowding, long waiting time, and short consulting time with doctors will lead to poor patient experience in eastern region.

In addition, our finding that indicated urban individuals with high education level was associated with poor patient experience is in line with previous study [56], mainly for the reason that less well-educated patients have much lower standards in evaluating the healthcare experience. Asymmetric information exits between patients and physicians in healthcare delivery procedure, and patients with high education level are more likely to get information about disease. With more independence, autonomy and decision-making built by person well-educated, the agreements in treatment and medication with physicians are possible to be influenced, leading to poor experience [58]. As for why such relationship doesn’t apply to rural counterparts, it may be accounted for a little education gap deriving from low education resources in rural area.

Our study supplements and improves the existing literatures on patient experience in the context of urban-rural dual structure in China from the perspective of residents. To the best of our knowledge, the key advantage of this study was a nationwide survey to explore the association between place of residence and patient experience with CEM application to balance the rural and urban two comparison groups. However, the findings should be interpreted with caution to some extent because of the following several limitations. First of all, there was a sample loss during preprocessing the data with CEM, which might make some estimates lack precision and affect the representativeness of demographics distribution. Secondly, due to the lack information of healthcare institutions respondents visited (i.e., grade, location and resources), the characteristics of hospitals that might influence patient experience couldn’t be well controlled when comparing patient experiences for rural and urban residents. Thus, it needs to be further examined in future studies. Thirdly, since data on multidimensional measures of patient experience were unavailable, such as waiting/consultation time, communication and interaction with doctors and nurses, and hospital environment, the patient experience bias might exist in our study. Fourthly, considering the targeted people in this study were residents instead of outpatients or inpatients, the results might be less accurate in measuring patient experience compared to patients investigated after their encounters with physicians.

## Conclusions

As found in this study, patient experience differences did exist in rural and urban residents in China. Rural residents had better patient experience than their urban counterparts. Older and healthier residents living in western area with higher social status, evaluation on adequacy, distribution and accessibility of healthcare resources were associated with better patient experience. In addition, urban residents with lower education and higher family economic status reported better patient experience. It is essential to not only encourage residents to form a habit of seeking healthcare services in local primary healthcare institutions first and then go to large hospitals in urban areas when necessary, but also abridge the disparity of healthcare resources between rural and urban areas by improving the quality and capacity of rural healthcare institutions and primary healthcare system of China.

## Supplementary Information

**Additional file 1.**

## Data Availability

The data that support the findings of this study are openly available in CGSS website: http://cgss.ruc.edu.cn.

## Abbreviation

CEM: Coarsened exact matching

## References

[1] Yan Z, Wan D (2011). Patient satisfaction in two Chinese provinces: rural and urban differences. Int J Qual Health Care.

[2] Manary MP, Boulding W, Staelin R, Glickman SW (2013). The patient experience and health outcomes. N Engl J Med.

[3] Black N, Jenkinson C (2009). Measuring patients’ experiences and outcomes. BMJ.

[4] Ahmed F, Burt J, Roland M (2014). Measuring patient experience: concepts and methods. Patient..

[5] Kemp KA, Santana MJ, Southern DA, McCormack B, Quan H (2016). Association of inpatient hospital experience with patient safety indicators: a cross-sectional, Canadian study. BMJ open.

[6] Elliott MN, Cohea CW, Lehrman WG, Goldstein EH, Cleary PD, Giordano LA, Beckett MK, Zaslavsky AM (2015). Accelerating improvement and narrowing gaps: trends in Patients' experiences with hospital care reflected in HCAHPS public reporting. Health Serv Res.

[7] Jenkinson C, Coulter A, Bruster S (2002). The picker patient experience questionnaire: development and validation using data from in-patient surveys in five countries. Int J Qual Health Care.

[8] Giordano LA, Elliott MN, Goldstein E, Lehrman WG, Spencer PA (2009). Development, implementation, and public reporting of the HCAHPS survey. Med Care Res Rev.

[9] Wiig S, Storm M, Aase K, Gjestsen MT, Solheim M, Harthug S (2013). Investigating the use of patient involvement and patient experience in quality improvement in Norway: rhetoric or reality?. BMC Health Serv Res.

[10] Doyle C, Lennox L, Bell D (2013). A systematic review of evidence on the links between patient experience and clinical safety and effectiveness. BMJ Open.

[11] Anhang Price R, Elliott MN, Zaslavsky AM, Hays RD, Lehrman WG, Rybowski L, Edgman-Levitan S, Cleary PD (2014). Examining the role of patient experience surveys in measuring health care quality. Med Care Res Rev.

[12] Mohammed K, Nolan MB, Rajjo T, Shah ND, Prokop LJ, Varkey P, Murad MH (2016). Creating a patient-centered health care delivery system: a systematic review of health care quality from the patient perspective. Am J Med Qual.

[13] Bao Y, Fan G, Zou D, Wang T, Xue D. Patient experience with outpatient encounters at public hospitals in Shanghai: examining different aspects of physician services and implications of overcrowding. PloS One. 2017;12(2):e0171684-e. 10.1371/journal.pone.0171684 PMC5312958 28207783

[14] Robert G, Cornwell J (2013). Rethinking policy approaches to measuring and improving patient experience. J Health Serv Res Policy.

[15] Tefera L, Lehrman WG, Conway P (2016). Measurement of the patient experience: clarifying facts, myths, and approaches. JAMA..

[16] Liu C, Wu Y, Chi X (2017). Relationship preferences and experience of primary care patients in continuity of care: a case study in Beijing, China. BMC Health Serv Res.

[17] Wang X, Jiang R, Li J, Chen J, Burström B, Burström K (2018). What do patients care most about in China’s public hospitals? Interviews with patients in Jiangsu Province. BMC Health Serv Res.

[18] Lu C, Hu Y, Xie J, Fu Q, Leigh I, Governor S, Wang G (2018). The use of Mobile health applications to improve patient experience: cross-sectional study in Chinese public hospitals. JMIR Mhealth Uhealth.

[19] Hu G, Chen Y, Liu Q, Wu S, Guo J, Liu S, Wang Z, Zhao P, Sun J, Hu L, Zhou H, Luo L, Mao Y, Needleman J, Ma J, Liu Y (2019). Patient experience of hospital care in China: major findings from the Chinese patient experience questionnaire survey (2016–2018). BMJ Open.

[20] Spasojevic N, Vasilj I, Hrabac B, Celik D (2015). Rural-urban differences in health care quality assessment. Mater Sociomed.

[21] Yaya S, Bishwajit G, Ekholuenetale M, Shah V, Kadio B, Udenigwe O (2017). Urban-rural difference in satisfaction with primary healthcare services in Ghana. BMC Health Serv Res.

[22] Gu H, Kou Y, You H, Xu X, Yang N, Liu J, Liu X, Gu J, Li X (2019). Measurement and decomposition of income-related inequality in self-rated health among the elderly in China. Int J Equity Health.

[23] Haggerty JL, Roberge D, Lévesque J-F, Gauthier J, Loignon C (2014). An exploration of rural-urban differences in healthcare-seeking trajectories: implications for measures of accessibility. Health Place.

[24] De la Cruz-Sánchez E, Aguirre-Gómez L (2014). Health related lifestyle and preventive medical Care of Rural Spanish Women Compared to their urban counterparts. J Immigr Minor Health.

[25] Fadum EA, Stanley B, Rossow I, Mork E, Törmoen AJ, Mehlum L (2013). Use of health services following self-harm in urban versus suburban and rural areas: a national cross-sectional study. BMJ Open.

[26] Oladipo JA (2014). Utilization of health care services in rural and urban areas: a determinant factor in planning and managing health care delivery systems. Afr Health Sci.

[27] Li J, Shi L, Liang H, Ding G, Xu L (2018). Urban-rural disparities in health care utilization among Chinese adults from 1993 to 2011. BMC Health Serv Res.

[28] Liu X, Li N, Liu C, Ren X, Liu D, Gao B, Liu Y (2016). Urban-rural disparity in utilization of preventive care services in China. Medicine (Baltimore).

[29] Chen Y, Yin Z, Xie Q (2014). Suggestions to ameliorate the inequity in urban/rural allocation of healthcare resources in China. Int J Equity Health.

[30] Zhang X, Dupre ME, Qiu L, Zhou W, Zhao Y, Gu D (2017). Urban-rural differences in the association between access to healthcare and health outcomes among older adults in China. BMC Geriatr.

[31] Li LW, Liu J, Xu H, Zhang Z (2016). Understanding rural-urban differences in depressive symptoms among older adults in China. J Aging Health.

[32] Fei X, Wu J, Kong Z, Christakos G (2015). Urban-rural disparity of breast Cancer and socioeconomic risk factors in China. PLoS One.

[33] Wang R, Chen Z, Zhou Y, Shen L, Zhang Z, Wu X (2019). Melancholy or mahjong? Diversity, frequency, type, and rural-urban divide of social participation and depression in middle- and old-aged Chinese: a fixed-effects analysis. Soc Sci Med.

[34] Wang S, Kou C, Liu Y, Li B, Tao Y, D'Arcy C (2015). Rural-urban differences in the prevalence of chronic disease in Northeast China. Asia Pac J Public Health.

[35] Aday L, Andersen R (1974). A framework for the study of access to medical care. Health Serv Res.

[36] Andersen R (1995). Revisiting the behavioral model and access to medical care: does it matter?. J Health Soc Behav.

[37] Andersen R, Newman JF (2005). Societal and individual determinants of medical care utilization in the United States. Milbank Quarterly.

[38] Iacus SM, King G, Porro G (2011). Multivariate matching methods that are monotonic imbalance bounding. J Am Stat Assoc.

[39] Iacus SM, King G, Porro G (2012). Causal inference without balance checking: coarsened exact matching. Polit Anal.

[40] Rubin DB, Rubin DB (2006). The use of matched sampling and regression adjustment to remove Bias in observational studies. Matched sampling for causal effects.

[41] Hametner C, Kellert L, Ringleb PA (2015). Impact of sex in stroke thrombolysis: a coarsened exact matching study. BMC Neurol.

[42] Condelius A, Andersson MJBG (2015). Exploring access to care among older people in the last phase of life using the behavioural model of health services use: a qualitative study from the perspective of the next of kin of older persons who had died in a nursing home. BMC Geriatr.

[43] Babitsch B, Gohl D, von Lengerke T (2012). Re-revisiting Andersen's behavioral model of health services use: a systematic review of studies from 1998–2011. Psychosoc Med.

[44] Cabrera-Barona P, Blaschke T, Kienberger S (2017). Explaining accessibility and satisfaction related to healthcare: a mixed-methods approach. Soc Indic Res.

[45] Niu G, Zhao G (2018). Survey data on political attitudes of China′s urban residents compiled from the Chinese general social survey (CGSS). Data Brief.

[46] Tetteh J, Kogi R, Yawson AO, Mensah G, Biritwum R, Yawson AE (2019). Effect of self-rated health status on functioning difficulties among older adults in Ghana: coarsened exact matching method of analysis of the World Health Organization's study on global AGEing and adult health, wave 2. PLoS One.

[47] Muennig P, Masters R, Vail D, Hakes J (2017). The effects of New York City's coordinated public health programmes on mortality through 2011. Int J Epidemiol.

[48] Green MA, Subramanian SV, Vickers D, Dorling D (2015). Internal migration, area effects and health: Does where you move to impact upon your health?. Soc Sci Med.

[49] China TCPsGo. Opinions of the State Council of the CPC Central Committee on deepening health system reform 2009 [cited 2020 2020-03-08]. Available from: http://www.gov.cn/test/2009-04/08/content_1280069.htm.

[50] Chen Z (2009). Launch of the health-care reform plan in China. Lancet.

[51] Yip W, Hsiao W (2014). Harnessing the privatisation of China's fragmented health-care delivery. Lancet.

[52] Pan J, Liu GG, Gao C (2013). How does separating government regulatory and operational control of public hospitals matter to healthcare supply?. China Econ Rev.

[53] Liu Y, Zhong L, Yuan S, van de Klundert J (2018). Why patients prefer high-level healthcare facilities: a qualitative study using focus groups in rural and urban China. BMJ Glob Health.

[54] Barber SL, Borowitz M, Bekedam H, Ma J (2013). The hospital of the future in China: China’s reform of public hospitals and trends from industrialized countries. Health Policy Plan.

[55] NHaFPCo C. (2016). Health and family planning statistics yearbook (2016).

[56] van der Veer SN, Arah OA, Visserman E, Bart HAJ, de Keizer NF, Abu-Hanna A, Heuveling LM, Stronks K, Jager KJ (2012). Exploring the relationships between patient characteristics and their dialysis care experience. Nephrol Dial Transpl.

[57] Hills R, Kitchen S (2007). Development of a model of patient satisfaction with physiotherapy. Physiother Theory Pract.

[58] Hu Y, Zhang Z (2015). Patient education – a route to improved patient experience in Chinese hospitals?. Patient Educ Couns.

