# Supplementary material for: Rural and urban differences in patient experience in China: a coarsened exact matching study from the perspective of residents
Source: BMC Health Serv Res. 2021 Apr 13;21:330. doi: 10.1186/s12913-021-06328-0 (PMC8042990; doi:10.1186/s12913-021-06328-0)
Supplement: Supplementary file 1 — Additional file 1. [file 12913_2021_6328_MOESM1_ESM.docx]

**Additional file**

Table S1 Definitions/codes of variables

| Type | | Variables | Definitions/codes |
| --- | --- | --- | --- |
| Dependent variable |  | Patient experience^1^ | Continuous variable (0-100) |
|  |  | Patient experience^2^ | Very bad=1 |
|  |  |  | Bad=2 |
|  |  |  | Fair=3 |
|  |  |  | Good=4 |
|  |  |  | Very good=5 |
| Independent variables | Predisposing factors | Age | 45 years and below=1 |
|  |  |  | 46-60 years=2 |
|  |  |  | above 60 years=3 |
|  |  | Gender | Male=0 |
|  |  |  | Female=1 |
|  |  | Education status | Primary school and below=1 |
|  |  |  | Junior and senior school=2 |
|  |  |  | College degree and above=3 |
|  |  | Marital status | Unmarried/Divorced/Widowed/Cohabiting=0 |
|  |  |  | Married=1 |
|  |  | Social status | Continuous variable (1-10) |
|  |  | Place of residence | Rural=0 |
|  |  |  | Urban=1 |
|  |  | Region | Eastern=1 |
|  |  |  | Middle=2 |
|  |  |  | Western=3 |
|  | Enabling factors | Personal income | Poorest=1 |
|  |  |  | 2nd=2 |
|  |  |  | Middle=3 |
|  |  |  | 4st=4 |
|  |  |  | Rishest=5 |
|  |  | Family economic status | Lower than average=1 |
|  |  |  | Average=2 |
|  |  |  | Higher than average=3 |
|  |  | Medical insurance | No=0 |
|  |  |  | Yes=1 |
|  |  | Evaluation on adequacy of healthcare resources | Inadequate=1 |
|  |  |  | Fair=2 |
|  |  |  | Adequate=3 |
|  |  | Evaluation on distribution of healthcare resources | Unbalanced=1 |
|  |  |  | Fair=2 |
|  |  |  | Balanced=3 |
|  |  | Evaluation on accessibility of healthcare resources | Inconvenient=1 |
|  |  |  | Fair=2 |
|  |  |  | Convenient=3 |
|  | Need factors | Self-rated health status | Unhealthy=1 |
|  |  |  | Fair=2 |
|  |  |  | Healthy=3 |

Note: ^1^ Measured by numerical score of evaluation on health services.

^2^ Measured by ordinal categorical of evaluation on health services.

Table S2 The association between place of residence and patient experience in the unmatched cohort with sample weights

| Variables | Crude analysis | |  | Multivariate analysis | |
| --- | --- | --- | --- | --- | --- |
|  | Coef. | OR |  | Coef. | OR |
|  | 95%CI | 95%CI |  | 95%CI | 95%CI |
| Place of residence (Ref: Rural) |  |  |  |  |  |
| Urban | -5.147^***^ | 0.577^***^ |  | -4.410^***^ | 0.627^***^ |
|  | (-5.967 - -4.326) | (0.529- 0.630) |  | (-5.309 - -3.511) | (0.565 - 0.695) |
| Control variables | No | No |  | Yes | Yes |

Note: Coef. means Coefficient. OR means Odds Ratio. 95%CI means 95% Confidence Interval.

^*^ *p*<0.05, ^**^ *p*<0.01, ^***^ *p*<0.001

^1^ Crude ordinary least-squares regression.

^2^ Crude ordered logistic regression.

^3^ Ordinary least-squares regression within the control of age, gender, education, marital status, social status, region, personal income, family economic status, medical insurance, the evaluation on adequacy, distribution and accessibility of healthcare resources, and self-rated health status.

^4^ Ordered logistic regression within the control of age, gender, education, marital status, social status, region, personal income, family economic status, medical insurance, the evaluation on adequacy, distribution and accessibility of healthcare resources, and self-rated health status.
